# Evolutionarily-conserved MZIP2 is essential for crossover formation in mammalian meiosis

**DOI:** 10.1038/s42003-018-0154-z

**Published:** 2018-09-21

**Authors:** Qianting Zhang, Jingchen Shao, Heng-Yu Fan, Chao Yu

**Affiliations:** 1 Department of Chemistry and Molecular Biology, University of Gothenburg, SE-40530 Gothenburg, Sweden; 2 Life Sciences Institute, Zhejiang University, Hangzhou, 310058 China

## Abstract

During meiosis, formation of crossovers—the physical links that ensure the segregation of homologous chromosomes—requires a group of evolutionarily conserved ZMM proteins. In budding yeast, three ZMM proteins, Zip2, Spo16, and Zip4, form a trimeric complex to bind recombination intermediates and promote crossover formation. Here, we show that MZIP2 is the mammalian ortholog of Zip2. Complete ablation of MZIP2 in mice caused sterility in both males and females, as well as defects in repairing meiotic DNA double-strand breaks. MZIP2 forms discrete foci on chromosomes axes, and is required for the localization of TEX11 (mammalian Zip4 ortholog) and another ZMM protein, MSH4, to form crossover-prone recombination intermediates. As a consequence, formation of crossovers is abolished and formation of synaptonemal complex is incomplete in MZIP2-null meiocytes, resulting in meiosis arrest at a zygotene-like stage. Our results suggest that the processing of early recombination intermediates toward mature crossovers is dependent on MZIP2.

## Abstract (web summary)

Qianting Zhang et al. characterized the role of the mammalian ortholog of Zip2 in meiotic crossovers. Loss-of-function of MZIP2 leads to defects in DNA double-strand break repair, halting meiosis and causing sterility in both male and female mice.

## Introduction

Eukaryotic meiosis is a specialized type of cell division that reduces ploidy through the segregation of homologous chromosomes (homologs), and renders a plenty of genetic diversity through homologous recombination and subsequent fertilization. Crossovers, which are formed between homologs in meiotic prophase I, are the physical links to render the connectivity and enable precise segregation of homologs at anaphase I. From yeast to humans, processing of early recombination intermediates, or D-loops, to mature crossovers is highly dependent on a group of functionally related proteins collectively known as ZMM or SIC (synapsis initiation complex)^1–3^.

The ZMM proteins were first identified in budding yeast, the deletion of which caused defects in formation of crossovers and synaptonemal complex, resulting in zipper-like pairing of homologs^1^. These proteins are: the transverse filament protein Zip1, assembling the central element of synaptonemal complex^4^; Zip2 and Spo16, forming an XPF-ERCC1-like complex^5–8^; Zip3, an E3 ligase^9^; Zip4, which interacts with Zip2–Spo16 complex^7,8,10^; a 5′–3′ DNA helicase, Mer3 (ref. ^11^); and the MutSγ components, Msh4 and Msh5 (refs. ^12,13^). During yeast meiosis, ZMM proteins localize to recombination intermediates shortly after strand invasion, and assemble two subcomplexes (Zip2–Zip4–Spo16 complex and Msh4–Msh5 complex) to control crossover interference (crossovers are properly distributed) and assurance (one homolog receive at least one crossover)^6^.

The functions of ZMM proteins in meiotic recombination are evolutionarily conserved. SYCP1 is believed to be orthologous to Zip1 based on its function in assembling the central element of synaptonemal complex, although they share less similarity in protein sequence^14^. Msh4, Msh5, and Mer3 are highly conserved from bacteria to humans, and their orthologs in mammals (MSH4, MSH5, and HFM1, respectively) and plants are well characterized. RNF212 and HEI10 have domain similarity with Zip3, and regulate the stability of the MSH4–MSH5 complex by ubiquitination and SUMOylation to designate crossovers and non-crossovers^15,16^. TEX11 is the mammalian ortholog of Zip4, loss-of-function of which causes infertility in both humans and mice^17–19^. Several recent studies suggest that Zip2, Zip4, and Spo16 form a trimeric complex to participate in crossover formation^7,8^. However, the mammalian orthologs of Zip2 and Spo16 in mammals remain elusive.

*C9orf84* was previously identified as the ortholog of *Saccharomyces cerevisiae* Zip2 and *Arabidopsis thaliana* SHOC1, based on the domain similarities^8,20^. In this study, we characterized the in vivo functions of protein encoded by *C9orf84*, namely mammalian Zip2 (MZIP2), in mammalian meiosis. Similar to the phenotypes reported in yeast *zip2* mutants, severe defects in meiotic prophase I, such as DNA double-strand break (DSB) repair, crossover formation, and synapsis, were observed in MZIP2-deleted spermatocytes and oocytes, resulting in sterility in both male and females. MZIP2 is required for the localization of other ZMM proteins, such as TEX11 and MSH4, to assemble the crossover-prone recombination intermediates. We suggest that MZIP2 facilitates the formation of ZMM proteins-associated recombinational intermediates to promote crossing-over in meiotic prophase I.

## Results

### Identification of mammalian ZIP2

Previous studies suggested that the protein encoded by the human gene *C9orf84* might be the ortholog of *S. cerevisiae* Zip2 and *A. thaliana* SHOC1, based on its domain similarities^8,20^. These proteins have a conserved XPF endonuclease-like central domain and a helix-hairpin-helix (HhH^2^) domain close to the C-termini, although they vary in length^7,8^. By forming an XPF-ERCC1-like complex, Zip2–Spo16 complex in budding yeast and SHOC1–PTD complex in thale cress bind and stabilize joint molecules to promote crossing-over during meiotic recombination^6,8,21^. In a previous study, *C9orf84* was identified as being specifically expressed in the meiotic female primordial germ cells in humans, suggesting possible involvement of C9ORF84 in meiotic prophase I^22^. Thus we named the proteins encoded by *C9orf84* and its orthologs in vertebrates MZIP2, and the genes, *Mzip2*. MZIP2 orthologs are found in vertebrates (68.4% consensus in amino acids from tropical clawed frog to human). Especially within the XPF-like central domain and the HhH^2^ domain, MZIP2 is highly conserved (92.7% consensus; Supplementary Fig. 1).

**Fig. 1:**
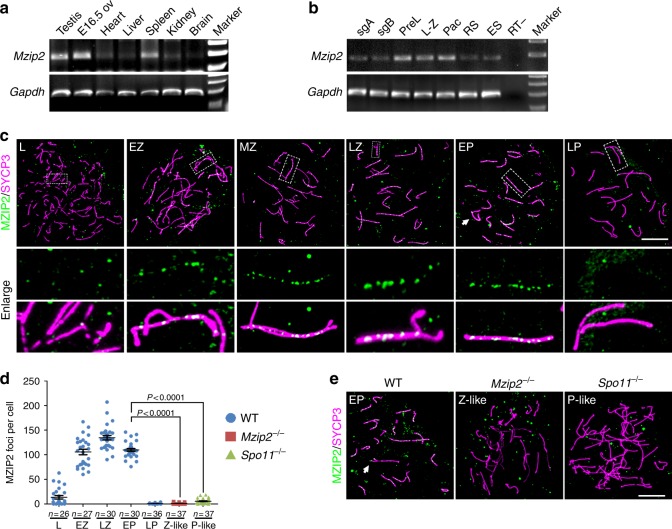
MZIP2 is specifically expressed in meiotic prophase I and localizes to chromosome axes. **a**, **b** PCR amplification to determine the relative expression level of *Mzip2* mRNA in multiple mouse tissues (**a**) and in male germ cells at different developmental stages (**b**). PCR results of *Gapdh* is served as a loading control. E16.5 ov ovaries at E16.5, sgA spermatogonia type A, sgB spermatogonia type B, PreL pre-leptonema, L–Z leptonema to zygonema, Pac pachynema, RS round spermatids, ES elongated spermatids, RT– without reverse transcriptase, negative control. **c** Immunofluorescent staining of MZIP2 and SYCP3 on nuclear surface spreads of wild-type spermatocytes at indicated stages. SYCP3 marked the meiotic chromosome axes. White arrow indicates XY body. Enlarged images showed the partially or fully synapsed homologous pairs, and the regions of which were bordered with dashed lines. L leptonema, EZ early-zygonema, MZ mid-zygonema, LZ late-zygonema, EP early pachynema, LP late-pachynema. Scale bar, 10 μm. **d** Quantification of MZIP2 foci detected on the chromosomes of WT, *Mzip2*^*−/−*^, and *Spo11*^*−/−*^ spermatocytes at indicated stages. Numbers of spermatocytes analyzed (*n*) were indicated. Z-like zygonema-like, P-like pachynema-like. Median focus numbers were marked. Error bar indicated S.E.M. *P*-values were assessed by unpaired two-tailed Student’s *t*-tests. **e** Immunofluorescent staining of MZIP2 on nuclear surface spreads of WT, *Mzip2*^*−/−*^, and *Spo11*^*−/−*^ testes. White arrow indicates XY body. Scale bar, 10 μm

*Ai481877* is predicted to be the mouse ortholog of *Mzip2*. Mouse *Mzip2* is located on chromosome 4 and is predicted to be composed of 28 exons, encoding a protein 1481 amino acids long. Amplification and sequencing of *Mzip2* fragments from testis cDNA samples confirmed the predicted sequence. By PCR amplification of cDNA samples derived from multiple mouse tissues, we confirmed that mouse *Mzip2* were detected in adult testes and embryonic ovaries at embryonic day 16.5 (E16.5), with one exception of lower expression in spleen samples (Fig. 1a). Moreover, the *Mzip2* mRNA level was increased in spermatocytes at pre-leptotene stage, when entering the meiotic prophase I (Fig. 1b).

To understand the localization of MZIP2 in mammalian meiosis, we generated an MZIP2 antibody (against amino acid 474–635) and analyzed its dynamics on chromosomes. As shown in Fig. 1c, d, MZIP2 foci were found as early as in leptonema spermatocytes (13.7 ± 3.52; *n* = 26), and the number of which was increased as the progression of meiotic recombination and formation of synaptonemal complex. The number of MZIP2 foci peaked around the late-zygotene stage (134.6 ± 4.41; *n* = 30), and slightly decreased to 109.8 ± 2.86 (*n* = 30) at the early-pachytene stage. However, MZIP2 foci were not found in late-pachynema spermatocytes. This localization pattern is similar to some of the known ZMM proteins, such as TEX11 (ref. ^18^). The specificity of MZIP2 antibody was confirmed by immunostaining in MZIP2-null spermatocytes (Fig. 1d, e). MZIP2 foci were not detected in *Spo11*^*−/−*^ spermatocytes that were arrested at a pachytene-like stage due to impaired generation of DSBs, suggesting that MZIP2 foci are specific to meiotic recombination (Fig. 1d, e).

### MZIP2-null mice are infertile

**Fig. 2:**
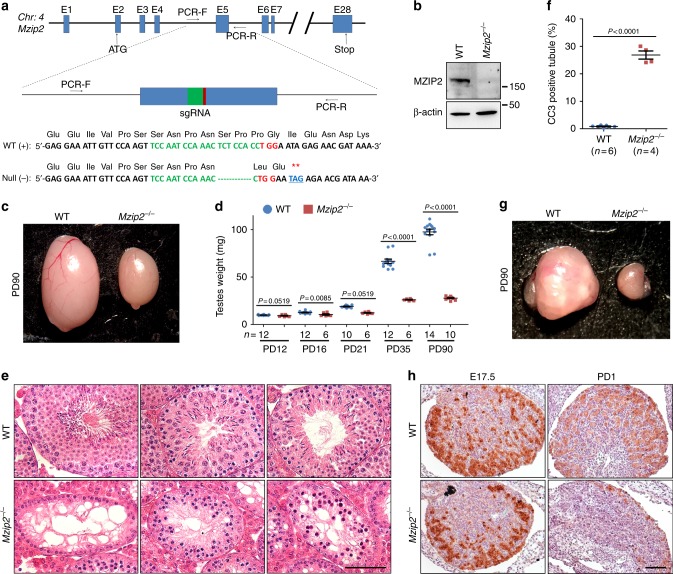
Deletion of MZIP2 led to infertility in both males and females. **a** Schematic diagram showing the gene structure of *Mzip2*, the targeting strategy, and the null allele. Exons, primers, sgRNA were indicated. The null allele had 7-bp deleted within the sgRNA region, resulting in a premature stop codon shortly after sgRNA (**). **b** Western blot results showing complete deletion of MZIP2 in testes. β-Actin level is served as a loading control. **c** A representative image showing smaller testes in *Mzip2*^*−/−*^ males at PD90. **d** The weights of testes derived from WT and *Mzip2*^*−/−*^ males at indicated ages. Numbers of testes analyzed (*n*) were indicated. Error bar indicated S.E.M. *P*-values were assessed by two-tailed Student’s *t*-tests. **e** H&E staining showing the histology of testes derived from WT and *Mzip2*^*−/−*^ males at PD42. Scale bars, 50 μm. **f** Percentage of CC3-positive seminiferous tubules in WT and *Mzip2*^*−/−*^ testes at PD21. **g** Morphology of WT and *Mzip2*^*−/−*^ ovaries at PD90. **h** Immunohistochemistry staining of MVH (mammalian homolog of vasa) showing oocytes in WT and *Mzip2*^*−/−*^ ovaries at E17.5 and PD1. Scale bar, 50 μm

To understand the functions of MZIP2 in mammalian meiosis, we designed a CRISPR/Cas9 strategy to generate null alleles for *Mzip2* (refs. ^23,24^). According to the strategy, a fragment on exon 5 was targeted by single-guide RNA (Fig. 2a). Through two repeats of microinjection and subsequent embryo transfer, five founders that contained certain mutations were identified by Sanger sequencing and were crossed with wild-type (WT) mice. Surprisingly, one founder male that was homozygous for an 8-bp insertion and one founder female that was homozygous for a 7-bp deletion were infertile when crossed with WT mice. We sacrificed the two mice and compared the testes or ovaries to the WT ones at the same age. As shown in Supplementary Fig. 2, the infertile male had dramatically smaller testes at postnatal day 70 (PD70), although few seminiferous tubules with round and elongated spermatids and residual sperm were observed on the sections stained with hematoxylin and eosin (H&E). The infertile female exhibited primordial follicle insufficiency, lacking oocytes or follicles at PD45 in the ovaries (Supplementary Fig. 2, b and d). Moreover, one null allele that had a 7-bp deletion was successfully transmitted to the germline and subsequent generations (Fig. 2a; Supplementary Fig. 3a, b). This allele introduced a premature stop codon shortly after the sgRNA and produced a residual peptide composed of the N-terminal 111 amino acids. This peptide should not be functional because the full-length protein has 1481 amino acids in total and the conserved XPF-like and HhH^2^ domains are located within amino acids 925–1156. Successful ablation of MZIP2 was confined by western blot with WT and *Mzip2*^*−/−*^ testes (Fig. 2b).

We further analyzed the knockout mice obtained from heterozygous to heterozygous breeding. The *Mzip2*^*−/−*^ mice were obtained according to the Mendelian ratio and the males had similar body size at different ages (Supplementary Fig. 3c; Supplementary Table 1), suggesting that MZIP2 is not required for the viability and development. However, both *Mzip2*^*−/−*^ males and females were infertile (Supplementary Table 2). Similar to the infertile founder male, *Mzip2*^*−/−*^ males had smaller testes, starting from the age of PD16, a stage by which the first wave of WT spermatocytes entered the pachytene stage (Fig. 2c, d). Adult *Mzip2*^*−/−*^ testes also had a size limitation of approximately 26 mg, resembling the testes of synapsis- and recombination-defective mouse models. H&E staining showed that *Mzip2*^*−/−*^ spermatocytes were arrested before the completion of meiotic prophase I, resulting in three kinds of seminiferous tubules: spermatogonia-only, prophase I arrested, and apoptotic (Fig. 2e; Supplementary Fig. 4a). Massive apoptosis was also examined by immunofluorescent staining of cleaved caspase 3 (CC3) on sections of WT and *Mzip2*^*−/−*^ testes at the age of PD21 (Fig. 2f; Supplementary Fig. 4b). However, the survival of undifferentiated spermatogonia and entry into meiosis were not affected by MZIP2 deletion (Supplementary Fig. 4c–e).

Similarly, germline loss is also observed in MZIP2-deleted females. Ovaries derived from *Mzip2*^*−/−*^ females at the age of PD90 were smaller than those of the WT controls (Fig. 2g). The oocytes in *Mzip2*^*−/−*^ ovaries were rapidly depleted between E17.5 and PD1, due to the defects in repairing meiotic DSBs (Fig. 2h; Supplementary Fig. 5a–c). No oocytes or follicles were observed in *Mzip2*^*−/−*^ ovaries from the age of PD6 (Supplementary Fig. 5d–f), suggesting a severe primordial follicle insufficiency phenotype in *Mzip2*^*−/−*^ females. Obesity was frequently observed in these MZIP2-deleted females at the age of 3 months, which might be an indirect effect of oocyte loss (Supplementary Fig. 3c). Taken together, these defects observed in meiotic prophase I demonstrated that MZIP2 is required for meiotic progression and for fertility in both males and females.

### MZIP2-null meiocytes were arrested at a zygotene-like stage

**Fig. 3:**
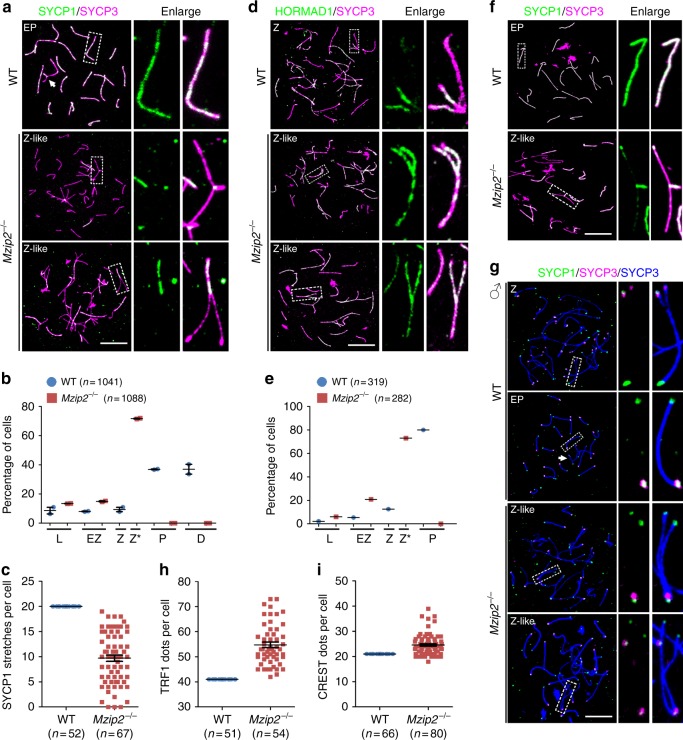
Meiocytes null for MZIP2 were arrested at a zygotene-like stage. **a** SYCP1 staining showing the zygotene-like stage of MZIP2-deleted spermatocytes. Z-like zygonema-like. White arrow indicates XY body. The regions bordered with dashed lines were enlarged on the right. Scale bar, 10 μm. **b** Meiotic progression of WT and *Mzip2*^*−/−*^ spermatocytes throughout meiotic prophase I. Numbers of spermatocytes analyzed (*n*) were indicated. Z zygonema, P pachynema, D diplonema, Z* zygotene-like. Error bars indicates S.E.M. **c** Quantification of SYCP1 stretches in WT pachynema spermatocytes and *Mzip2*^*−/−*^ zygonema-like spermatocytes. Numbers of spermatocytes analyzed (*n*) were indicated. Error bars indicated S.E.M. **d** HORMAD1 staining showing the zygotene-like stage of MZIP2-deleted spermatocytes. Scale bar, 10 μm. **e** Meiotic progression of oocytes derived from WT and *Mzip2*^*−/−*^ females at E17.5. Numbers of spermatocytes analyzed (*n*) were indicated. **f** SYCP1 staining on nuclear surface spreads of oocytes derived from WT and *Mzip2*^*−/−*^ females at E17.5. Scale bars, 10 μm. **g** Staining of a telomeric marker (TRF1) and a centromeric marker (CREST) on nuclear surface spreads of WT and *Mzip2*^*−/−*^ spermatocytes. White arrow indicates XY body. Scale bars, 10 μm. **h**–**i** Quantification of TRF1 and CREST foci on nuclear surface spreads of WT spermatocytes at a pachytene stage or MZIP2-deleted spermatocytes arrested at a zygotene-like stage

Synapsis is a marker of meiotic prophase I progression. During mammalian meiosis, the transverse filament protein SYCP1 assembles between the homologs and marks the regions of synapsis in zygonema and pachynema spermatocytes, and dissociates from the homologs after crossover formation in diplonema spermatocytes^14^. Therefore, in WT pachynema spermatocytes, SYCP1 overlapped with SYCP3, a lateral synaptonemal complex protein that marks the chromosome axes, except for the unsynapsed XY chromosomes (Fig. 3a). However, in *Mzip2*^*−/−*^ spermatocytes, SYCP1 initiated assembly from some regions, but failed to stretch to the full length between homologs, resulting in zipper-like forks (Fig. 3a). This is similar to the phenotypes of Zip2-null budding yeast, suggesting the evolutionarily conserved roles of MZIP2 in meiotic recombination^5^. We characterized this stage as zygotene-like and quantified the distribution of spermatocytes at different stages in WT and *Mzip2*^*−/−*^ testes. *Mzip2*^*−/−*^ spermatocytes never developed beyond the zygotene-like stage, resulting in reduced numbers of initiated SYCP1 stretches (Fig. 3b, c). The defects in synaptonemal complex formation were similarly examined by staining of SYCP1 on testis sections, and another marker of synapsis, SIX6OS1, in nuclear surface spreads (Supplementary Fig. 6a, b). HORMAD1, which localizes to the unsynapsed regions in meiotic prophase I, was largely retained in the zygotene-like *Mzip2*^*−/−*^ spermatocytes (Fig. 3d). We also prepared meiotic oocytes using embryonic ovaries and found that *Mzip2*^*−/−*^ oocytes were arrested at a similar zygotene-like stage without the possibility of full synapsis (Fig. 3e, f). When stained with a centromeric marker, CREST, and a telomeric marker, TRF1, the numbers of centromeres and telomeres were found to be increased in *Mzip2*^*−/−*^ spermatocytes, due to the impaired synapsis (Fig. 3g–i). Because the number of TRF1 foci was almost two-fold that of the CREST foci, we doubled the CREST foci for comparison with the increase in TRF1 foci. Interestingly, the latter was more dramatic than the former (20.0% vs. 33.5%; *P* < 0.0001, two-tailed *t*-test), indicating that homologs preferentially initiate pairing from the centromeres, at least in these *Mzip2*^*−/−*^ spermatocytes.

**Fig. 4:**
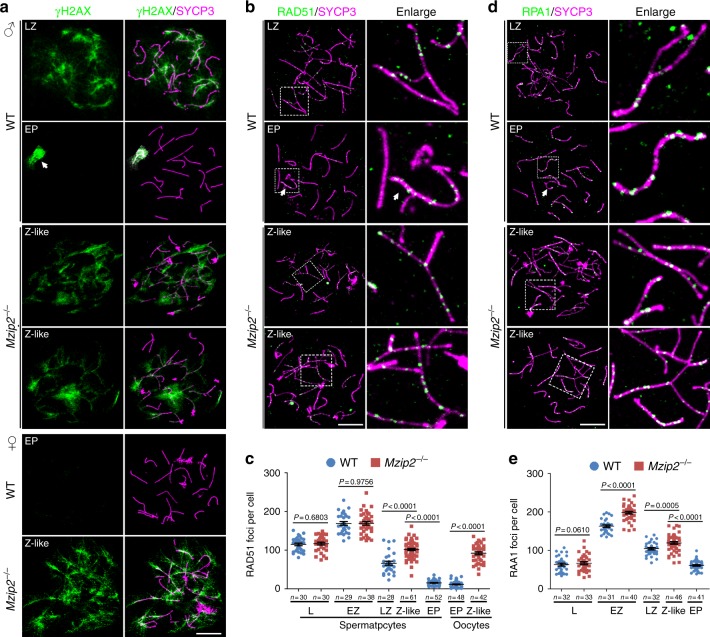
MZIP2-null meiocytes failed to repair meiotic DSBs. **a** γH2AX (green) staining on nuclear surface spreads of WT and *Mzip2*^*−/−*^ spermatocytes and primordial germ cells at indicated stages. White arrow indicates XY body. Scale bar, 10 μm. **b** Nuclear surface spreads of WT and *Mzip2*^*−/−*^ spermatocytes were stained with the early recombination marker, RAD51. White arrow indicates XY body. The regions bordered with dashed lines were enlarged on the right. Scale bar, 10 μm. **c** Quantification of RAD51 foci detected in WT and *Mzip2*^*−/−*^ spermatocytes or oocytes at indicated stages. **d**, **e** RPA1 was stained in WT and *Mzip2*^*−/−*^ spermatocytes at different stages (**d**), and the quantification of which was shown in **e**. White arrow indicates XY body. Scale bars, 10 μm

γH2AX is a marker of DNA double-strand breaks, representing the generation and repair of DSBs in meiotic prophase I. Therefore, in WT spermatocytes, γH2AX localized exclusively to the unpaired regions of XY chromosome pairs (or sex body) from the pachytene stage (Fig. 4a). However, in *Mzip2*^*−/−*^ testes, spermatocytes never reached the pachytene stage. γH2AX signal was distributed widely throughout the nuclei of these spermatocytes (Fig. 4a, Supplementary Fig. 6c), indicating the defects in DSB repair. Taken together, *Mzip2*^*−/−*^ meiocytes are arrested at a zygotene-like stage before the completion of meiotic recombination and DSB repair.

### Defects in meiotic recombination in MZIP2-null meiocytes

The defects in DSB repair and synapsis are indicative of the functions of MZIP2 in meiotic recombination. To dissect the processes of meiotic recombination in meiocytes null for MZIP2, we stained nuclear surface spreads with various markers of meiotic recombination. RAD51, a recombinase, functions in both meiotic and mitotic homologous recombination by binding to the resected single-stranded DNAs and mediating the formation of D-loops together with DMC1 (refs. ^25,26^). In WT spermatocytes, RAD51 first localized to single-stranded DNA at the leptotene stage, and was removed from these sites during the zygotene to pachytene transition (Fig. 4b; Supplementary Fig. 6d). At the early-pachytene stage, the number of RAD51 foci was reduced to approximately one-tenth compared to the early-zygotene stage (Fig. 4b). In *Mzip2*^*−/−*^ spermatocytes, RAD51 was similarly recruited to single-stranded DNA at the leptotene and early-zygotene stages, and gradually decreased from the early-zygotene stage to the zygotene-like stage (Fig. 4b, c; Supplementary Fig. 6d). However, the number of RAD51 foci in zygotene-like *Mzip2*^*−/−*^ spermatocytes was significantly higher than the one in late-zygotene and early-pachytene WT spermatocytes (*P* < 0.0001, two-tailed *t*-test; Fig. 4b, c). Similarly, there were more RAD51 foci in zygonema-like oocytes derived from the E17.5 *Mzip2*^*−/−*^ ovaries (Fig. 4c; Supplementary Fig. 6e).

A family of RPA proteins (replication protein A, RPA1/2/3) binds to single-stranded DNA overhangs during homologous recombination and DNA replication. Therefore, in meiotic prophase I, RPA1 and RPA2 localized to the early and middle recombination intermediates (Fig. 4d, e; Supplementary Fig. 7). The numbers of RPA1 and RPA2 foci increased along with the resection of DSBs in leptonema and peaked in early-zygonema spermatocytes, but decreased in late-zygonema and early pachynema after DSB repair (Fig. 4d, e; Supplementary Fig. 7a–c). In *Mzip2*^*−/−*^ spermatocytes, the numbers of RPA1 and RPA2 foci were similar to the WT controls at the leptotene and early-zygotene stages (Fig. 4d, e; Supplementary Fig. 7a–c), suggesting that the early processes of meiotic recombination are less affected by MZIP2 deletion. However, the zygonema-like *Mzip2*^*–/–*^ spermatocytes had comparable numbers of RPA1 and RPA2 foci as the WT late-zygonema spermatocytes, which was more than WT early-pachynema spermatocytes (Fig. 4d, e; Supplementary Fig. 7a–c). Moreover, staining of SPATA22, a meiosis-specific protein that interacts with RPA proteins^27,28^, showed the same pattern to RPA1 and RPA2 (Supplementary Fig. 7d–f). These results demonstrated that the DSBs generated in *Mzip2*^*−/−*^ meiocytes failed to be repaired through meiotic recombination.

### MZIP2 is required for ZMM foci formation and crossing-over

**Fig. 5:**
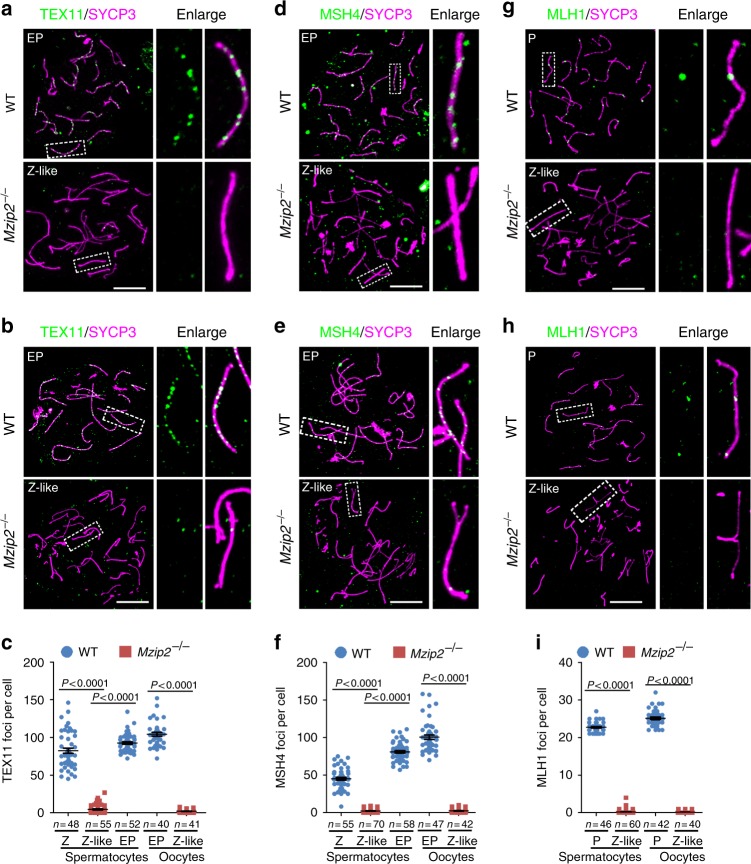
Defective meiotic recombination and crossing-over in MZIP2-deleted meiocytes. **a**–**c** Staining of TEX11 on nuclear surface spreads prepared with WT and *Mzip2*^*−/−*^ testes (**a**) or ovaries (**b**). The quantification of TEX11 foci was shown in **c**. Scale bars, 10 μm. **d**–**f** Staining for spermatocytes (**d**) and oocytes (**e**), and quantification (**f**) of MSH4 on the nuclear surface spreads. Scale bars, 10 μm. **g**–**i** MLH1 was stained to indicate late recombination intermediates in WT and MZIP2-deleted spermatocytes (**g**) or oocytes (**h**). The quantification of MLH1 foci was shown in **i**. Scale bars, 10 μm

As the next step to D-loop formation, ZMM proteins are recruited to a number of these recombination intermediates to promote the formation of crossover-prone joint molecules, SEIs and dHJs^1^. In budding yeast, Zip2 forms a trimeric complex with Spo16 and Zip4, or ZZS complex, which is recruited to the joint molecules for their stabilization during meiotic recombination^7,8^. TEX11, the mammalian homolog of Zip4, is required for female and male fertility in mice and humans^17–19^. TEX11 localized to the recombination sites in meiotic prophase I, and marked the crossover-prone recombination nodules in WT zygonema and early-pachynema spermatocytes or oocytes (Fig. 5a–c). Contrarily, TEX11 foci were not observed in zygonema-like *Mzip2*^*−/−*^ meiocytes (Fig. 5a–c), suggesting that MZIP2 is required for the localization of TEX11. Considering the fact that only Zip2 in the Zip2–Spo16–Zip4 (ZZS) complex possesses a known DNA-binding domain^8^, defects in TEX11 localization should be the direct effect of MZIP2 deletion. Besides the ZZS complex, Msh4 and Msh5 form another ZMM complex to promote crossover assurance and interference in budding yeast^6^. Similar to TEX11, MSH4 localized to the middle recombination intermediates in`WT early-pachynema meiocytes, and the recruitment of MSH4 was totally dependent on MZIP2 during mouse meiosis (Fig. 5d–f). Therefore, we propose that MZIP2 promotes meiotic recombination by recruiting other ZMM proteins to the recombination intermediates.

As a result, the crossover-specific late recombination nodules, which were indicated by anti-MLH1 staining, were never formed in either *Mzip2*^*−/−*^ spermatocytes or *Mzip2*^*−/−*^ oocytes that were arrested at the zygotene-like stage (Fig. 5g–i). Altogether, the phenotypes of *Mzip2*^*−/−*^ meiocytes in DSB repair, synapsis, dHJ formation, and crossing-over, suggested the conserved roles of MZIP2 in meiotic homologous recombination in meiotic prophase I.

## Discussion

**Fig. 6:**
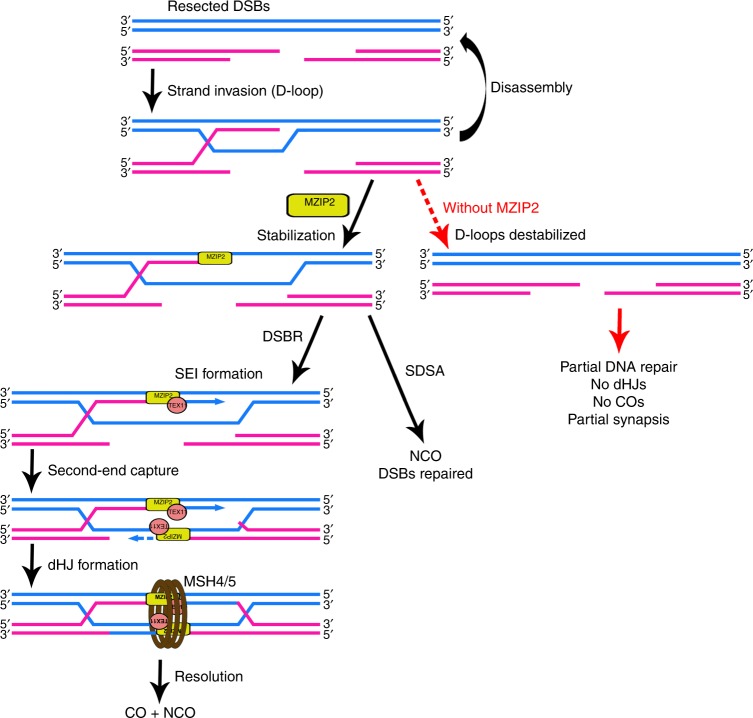
Schematic diagram showing the proposed function of MZIP2 in meiotic recombination. During meiotic recombination, MZIP2 is recruited to recombination intermediates and facilitates the assembly of ZMM foci, which function collectively to promote the formation of crossovers-related recombination intermediates. However in meiocytes null for MZIP2, D-loops are destabilized and remain unrepaired

A recent study has investigated the functions of mammalian Zip2 (called SHOC1 in that study) using a hypomorphic mouse model (*Mzip2*^*hyp/hyp*^), in which a truncated but functional MZIP2 is expressed in a relatively lower level^29^. As a result, *Mzip2*^*hyp/hyp*^ spermatocytes are arrested at metaphase I with a reduced rate of crossing-over (reduced MLH1 foci and univalent chromosomes at metaphase), whereas meiotic oocytes and female fertility are not affected. In this study, we generated a complete deletion mouse model of MZIP2 (Fig. 2b), which exhibited much stronger phenotypes in DSB repair, meiotic recombination, synapsis, and crossover formation, when compared to the hypomorphic model (Fig. 6, Supplementary Table 3). Strikingly, both *Mzip2*^*−/−*^ males and *Mzip2*^*−/−*^ females are sterile due to the consequent defects in meiotic recombination. Spermatoyctes null for MZIP2 are arrested at a zygotene-like stage without sufficient synapsis and DSB repair, which results in smaller testes than the hypomorphic model (Supplementary Table 3). Neither ZMM-associated middle recombination intermediates nor MLH1-associated late recombination intermediates are formed in MZIP2-deleted spermatocytes and oocytes. Therefore, we conclude that the roles of Zip2 orthologs in meiotic recombination are highly conserved in yeast, plants, and mammals. We provide a better model to understand the functions of MZIP2 in mammalian meiosis.

The XPF-like domain in Zip2 was recognized through a PSI-BLAST using an *At*SHOC1 homology domain, which identified a conserved domain among Zip2, SHOC1, and mammalian C9ORF84 (ref. ^20^). A recent study has determined the crystal structure of yeast Zip2 in complex with Spo16 (ref. ^7^). Similar to the known XPF-ERCC1-like heterodimers (MUS81–EME1/2 complexes and FANCM–FAAP24 complex), Zip2 and Spo16 form an XPF-ERCC1-like complex, in which Zip2 possesses an XPF-like domain to interact with DNA^30^. However, the residues constituting the active endonuclease site in XPF are not conserved in Zip2, resulting in inactive endonuclease activity. Based on biochemical experiments, recent studies suggest that the Zip2–Spo16 complex preferentially binds branched DNA structures that are frequently found in meiotic recombination, such as D-loops and Holliday junctions^8,29^. Therefore the authors proposed a model in which the Zip2–Spo16 complex binds and stabilizes early recombination intermediates to promote the formation of both crossovers and synaptonemal complex in meiotic prophase I. Based on these insights and our results, we suggest that MZIP2 is recruited to the early recombination intermediates shortly after D-loop formation, and stabilizes D-loops from being disassembled, through direct DNA–protein interaction or by promoting the extension of D-loops (Fig. 6). We infer that this step occurs prior to SEI formation (Fig. 6), because in yeast *zip2* mutants, the number of crossover-specific DNA intermediates, SEIs and dHJs, are dramatically reduced^31^. As a result, MZIP2-deleted meiocytes are arrested at a zygotene-like stage with fairly few attachments between homologous chromosomes and impaired DSB repair.

In budding yeast, the Zip2–Spo16 complex interacts with a third ZMM protein, Zip4, or Spo22 (refs. ^6–8^). Zip4 binds to the N-terminal regions of Zip2, while Spo16 interacts with the XPF-like domain close to the C-terminus, forming a trimeric ZZS complex that facilitates crossover formation, in at least two ways: (1) stabilization of D-loops, which is carried by Zip2–Spo16 subcomplex through protein–DNA interaction; and (2) recruitment of other ZMM proteins, possibly through interaction with Zip4 (ref. ^8^). Interestingly, the localization of TEX11, the mammalian ortholog of Zip4, and the component of the MutS complex, MSH4, is totally abolished upon MZIP2 deletion, suggesting that the recruitment of TEX11 and MSH4–MSH5 complex to the recombination intermediates depends on MZIP2. This is controversial to the early functions of Msh4–Msh5 complex in homolog coalignment during leptotene in *Sordaria macrospora*^32^. We infer that, at least in mammals, the ZZS complex functions in ahead of the MSH4–MSH5 complex in the processing of D-loops to dHJs.

Although the formation of crossovers is severely affected by deletion of MZIP2 and TEX11, it is noteworthy that the phenotypes in DSB repair and synapsis are divergent. While MZIP2-null spermatocytes are arrested at a zygotene-like stage with unrepaired DSBs and zipper-like asynapsis, major DSBs are repaired and full synapsis is achieved in spermatocytes null for TEX11, resulting in a metaphase I arrest^17,18,29^. Noting that TEX11 does not contain any known DNA-binding domain^7,8^, this difference might suggest that MZIP2 functions in ahead of TEX11 in meiotic recombination. Another possibility is that the original TEX11 mutants, similar to the mouse model expressing a hypomorphic MZIP2 allele, might not be complete deletion^17,18^. Interestingly, the association of TEX11 mutations with human infertility (non-obstructive azoospermia) is well characterized^19,33,34^. Splicing and missense mutations of *Tex11* are identified in infertile men with high possibilities (2.4–7.3%). It will be interesting to investigate the physiological functions of MZIP2 in human patients affected by non-obstructive azoospermia and premature ovarian insufficiency. Because meiosis phenotypes are also observed in the hypomorph MZIP2 model^29^, human fertility might be affected by *Mzip2* mutations that occur in exons, introns, promoter as well as enhancers.

However, the mammalian ortholog of Spo16 still remains elusive. Among the XPF-ERCC1-like complexes mentioned above, the cofactors, such as ERCC1, FAAP24, EME1/2, Spo16, and PTD, are less conserved. We failed to identify the mammalian Spo16 by BLAST with Spo16 and PTD against mammalian proteins. In some archaea, the XPF-like proteins function as a homodimer. This may not be the case for the mammalian Zip2–Spo16 complex, because in eukaryotic cells, the cofactors are more likely another protein containing an inactive nuclease domain^30^. It will be interesting to identify mammalian Spo16 to understand the functions of the Zip2–Spo16 complex in mammalian meiosis, or the possibility to increase homologous recombination efficiency by this complex during gene targeting.

## Methods

### Mice

Mice carrying null allele of *Spo11* were reported previously and were purchase from the Jackson Laboratory^35^. Null alleles for *Mzip2* was generated by CRISPR/Cas9 technology according to standard protocols^23,24,36^. A guide sequence of 5′-CCTAGAAAATCGAAGCCACA-3′ with a PAM sequence was selected based on the website prediction results (http://crispr.mit.edu/). The guide sequence was cloned to the pUC57-sgRNA vector. Following linearization, sgRNA and *Cas9* mRNA were in vitro transcribed and purified according to the manufacturer’s instructions (Ambion, AM1345, AM 1354, AM1908 and QIAGEN, 74104). Mouse zygotes were obtained by superovulation of 7-8-week-old females mating with males of the same strain. Mixture of *Cas9* mRNA (40 ng/μl) and sgRNA (40 ng/μl) was injected into zygotes by Eppendorf transferman NK2. Injected zygotes were transferred into pseudopregnant ICR female mice (15–25 zygotes per mouse) after 2 h recovery culture in KSOM medium.

All mice were maintained under SPF conditions in a controlled environment of 20–22 °C, with a 12/12 h light and dark cycle, 50–70% humidity, and food and water provided ad libitum. Animal care and experimental procedures were conducted in accordance with the University of Gothenburg, Sweden and Zhejiang University, China. All mutant mouse strains had a C57BL/6 background. The sexes and ages of mice used for specific experiments were indicated in figure legends. The images of testes and ovaries were taken under a stereomicroscope by a digital camera. Genotyping primers used are listed in Supplementary Table 4.

### Semi-quantitative reverse transcriptional PCR

Except for the embryonic ovary sample that was collected from embryonic females at E16.5, other tissues were from adult male mice. Male germ cells were prepared by a bovine serum albumin (BSA) gradient as previously described^37^. Total RNA was extracted using RNeasy Mini kit (Qiagen, #74106) according to the manufacturer’s instructions and reverse-transcribed to obtain cDNA (Bio-Rad, # 1708890). PCR was performed with Taq DNA polymerase under standard conditions (PCR for 28–32 cycles). *Gapdh* was served as a loading control. Primer sequences are listed in Supplementary Table 4, and uncropped gel images are shown in Supplementary Fig. 8.

### Western blotting

Testes were lysed directly in 2-mercaptoethanol containing sodium dodecyl sulfate (SDS) loading buffer and heated at 95 °C for 5 min. SDS-PAGE and immunoblots were performed following standard procedures using a semi-dry transfer system (Bio-Rad). The antibodies used are listed in Supplementary Table 5, and uncropped gel images are shown in Supplementary Fig. 8.

### Nuclear surface spreading

Seminiferous tubules were prepared from juvenile and adult testes, while female primordial germ cells were obtained from embryonic ovaries at E17.5. Nuclear surface spreads were prepared as previously described^38^. In brief, seminiferous tubules or embryonic ovaries were treated with a hypotonic buffer (30 mM Tris, 50 mM sucrose, 17 mM trisodium citrate dehydrate, 5 mM EDTA, and 0.5 mM DTT, pH 8.2) for 30 min, and subsequently smashed in 100 mM sucrose buffer (pH 8.2). The suspension was then added to a slides containing fixative buffer (1% paraformaldehyde and 0.15% Triton X-100, pH 9.2). After at least 2 h incubation in a humidify box, the slides were air dried and washed.

### Immunofluorescent staining and imaging

After extensive washing in phosphate-buffered saline (PBS), slides were blocked with 1% BSA in PBST (PBS with 0.1% tween-20) for 30 min, and subsequently subjected to incubation with primary antibodies and secondary antibodies. The primary antibodies used are listed in Supplementary Table 5, while the secondary antibodies with minimal cross-reactivity were purchased from JaxImmuno Research. The signals were examined under a DeltaVision microscope and images for quantification were taken by this microscope. Representative images were taken by a confocal laser scanning microscope (Zeiss LSM 700, Carl Zeiss AG, Germany). The foci of recombination proteins were quantified manually, and only foci found along chromosome axes were counted. For quantification of SYCP1 stretches, only the ones overlapped with SYCP3 were counted.

### Histological analyses

PBS-buffered formalin-fixed, paraffin-embedded samples were sectioned (5-μm-thick) for H&E staining and immunohistochemistry. To gain better histology of testis seminiferous tubules, testes were fixed in Bouin’s solution and subjected to H&E staining. Immunohistochemistry was performed as described previously^39^. Sections were deparaffinized and rehydrated. Primary antibodies were applied at suitable dilutions (Supplementary Table 5) at room temperature for 1 h, and then incubated with biotinylated secondary antibodies for 30 min. Sections were then stained using Vectastain ABC and DAB peroxidase substrate kits (Vector Laboratories, Burlingame, CA).

### Statistics

Results are given as means ± S.E.M. Each experiment included at least three independent samples or was repeated at least three times. Statistical analyses were performed by GraphPad Prism. Results for two experimental groups were compared by two-tailed unpaired Student’s *t*-tests. Statistically significant values (*P-*values) were indicated, when possible.

## Electronic supplementary material

Supplementary Information

## Data Availability

The datasets generated during the current study are available from the corresponding author on reasonable request.
