# Supplementary material for: Evolutionarily-conserved MZIP2 is essential for crossover formation in mammalian meiosis
Source: Commun Biol. 2018 Sep 21;1:147. doi: 10.1038/s42003-018-0154-z (PMC6155065; doi:10.1038/s42003-018-0154-z)
Supplement: Supplementary file 1 — Supplementary Information [file 42003_2018_154_MOESM1_ESM.pdf]

SUPPLEMENTARY FIGURES

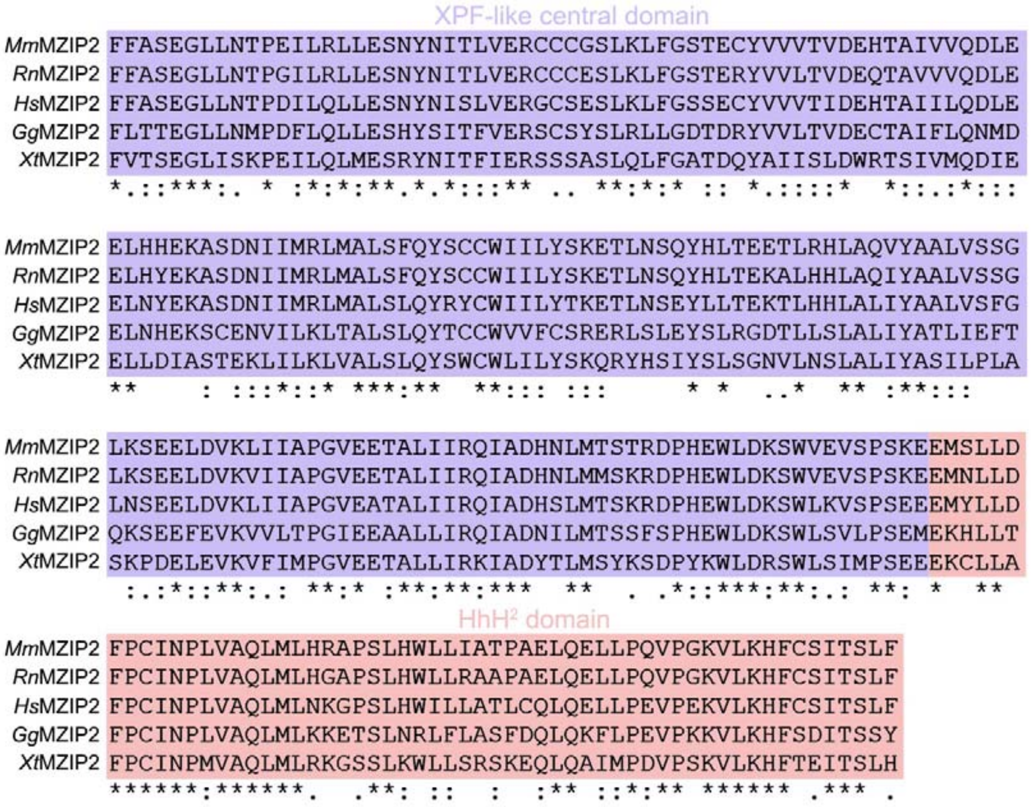

Supplementary Figure 1. ClustalW alignment results showing the consensus of the XPF-like central domains and helix-hairpin-helix (HhH^2) domains in MZIP2 from mouse (*Mm*), rat (*Rn*), human (*Hs*), tropical clawed frog (*Xt*) and chick (*Gg*). The two domains are highlighted in different colors.

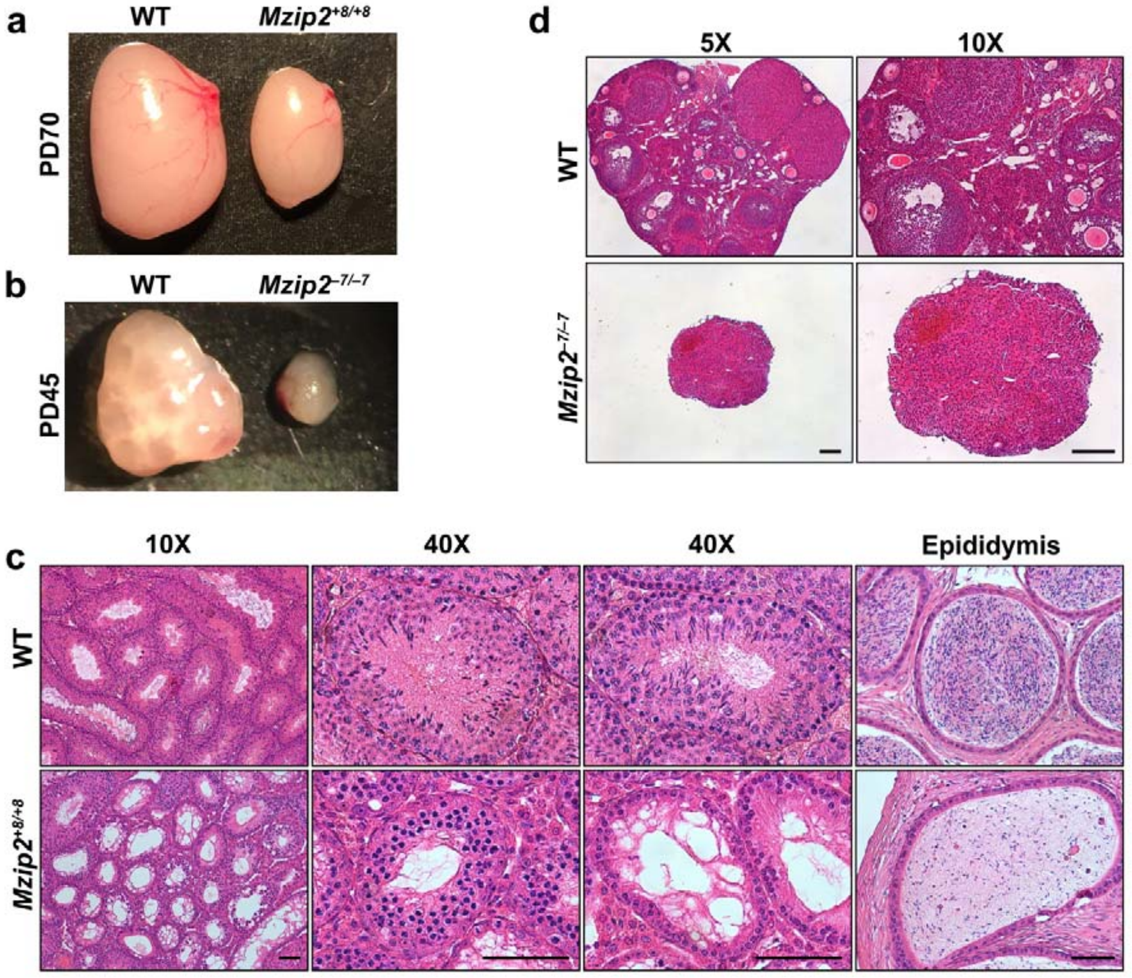

Supplementary Figure 2. CRISPR/Cas9-mediated knockout founder male and female. a, The infertile male that homozygous for a 8-bp insertion (*Mzip2*$^{+8/+8}$) had smaller testes at PD70. Only one mouse was analyzed in this experiment. **b,** The infertile female that homozygous for a 7-bp deletion (*Mzip2*$^{-7/-7}$) exhibited premature ovarian insufficiency (POI) at PD45. Only one mouse was analyzed. **c**, Mixed testicular atrophy in the testes derived from the founder *Mzip2*$^{+8/+8}$ male. Scale bars, 50 µm. **d**, No oocytes or follicles were observed in the founder *Mzip2*$^{-7/-7}$female. Scale bars, 100 µm.

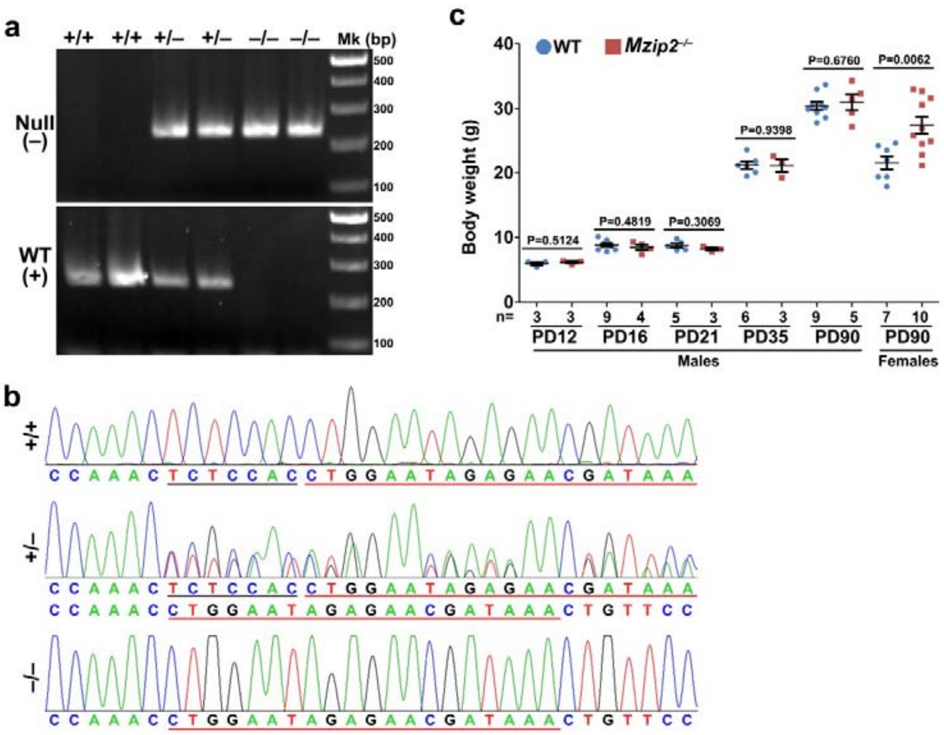

Supplementary Figure 3. Deletion of MZIP2 in mouse. a, Genotyping protocol to distinguish wild-type and null allele of *Mzip2*. **b**, Sequencing results of mice with different genotypes: *Mzip2*$^{+/+}$, *Mzip2*$^{+/–}$ and *Mzip2*$^{–/–}$. **c**, Body weights of WT and *Mzip2*$^{–/–}$ mice at indicated ages. Error bars indicated S.E.M. P values were assessed by two-tailed Student's *t*-tests.

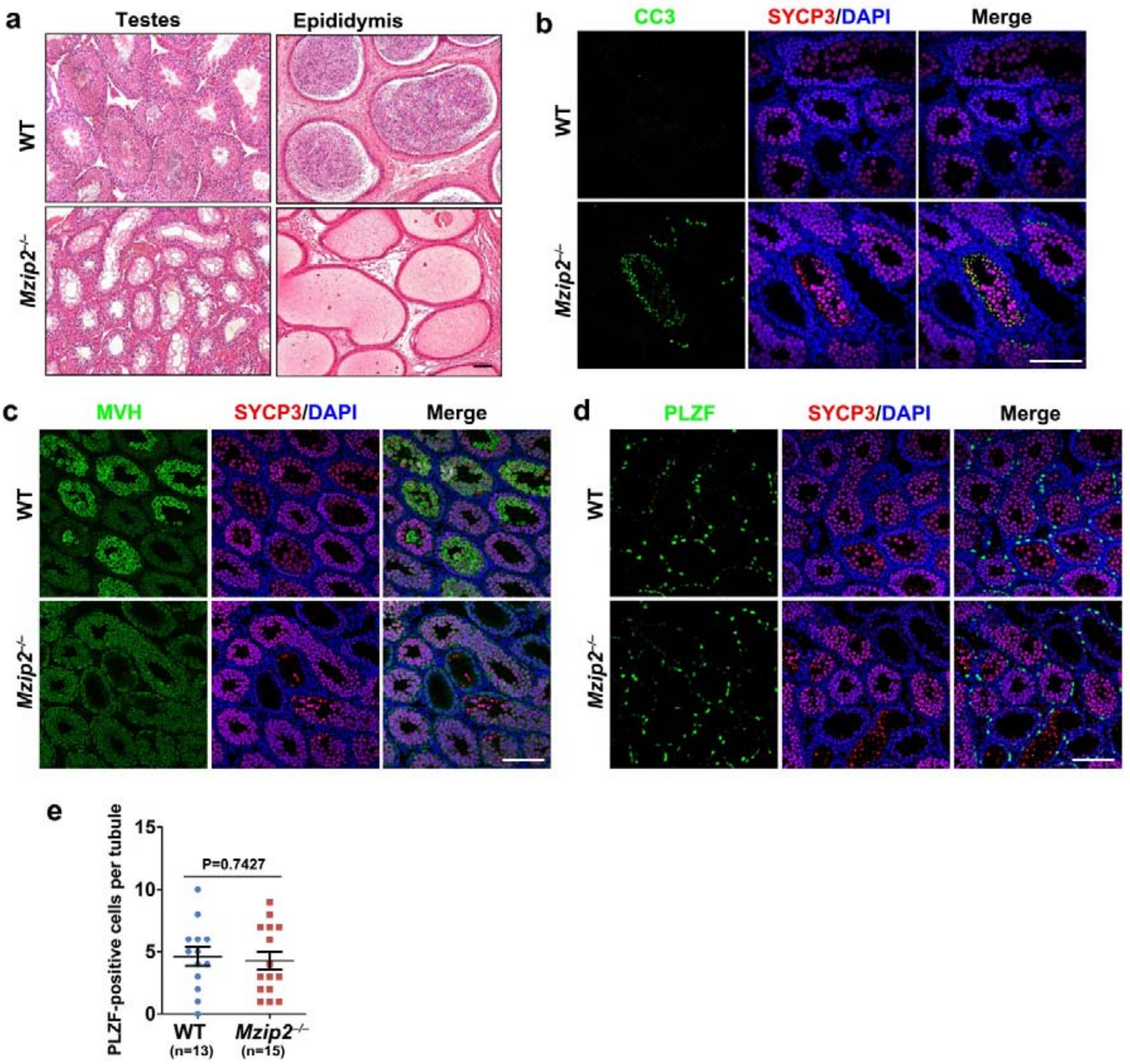

Supplementary Figure 4. Deletion of MZIP2 in males led to massive germ cell loss in testes. **a,** Hematoxylin and eosin (H&E) staining showing the histology of testes and epididymis derived from WT and *Mzip2*$^{-/-}$ males at PD42. Scale bars, 50 µm. **b,** Staining of cleaved caspase 3 (CC3) showing massive apoptosis in *Mzip2*$^{-/-}$ testes derived from PD21 males, and the quantification of CC3-positive seminiferous tubules was shown in Fig. 2f. Scale bar, 100 µm. **c–d**, Immunofluorescent staining of MVH (**c**) and PLZF (**d**) on sections of WT and *Mzip2*$^{-/-}$ testes at the age of PD21. Scale bars, 100 µm. **e**, Quantification of PLZF-positive cells in each tubule on sections of WT and *Mzip2*$^{-/-}$ testes. Error bar indicates S.E.M. Numbers of tubules quantified are indicated.

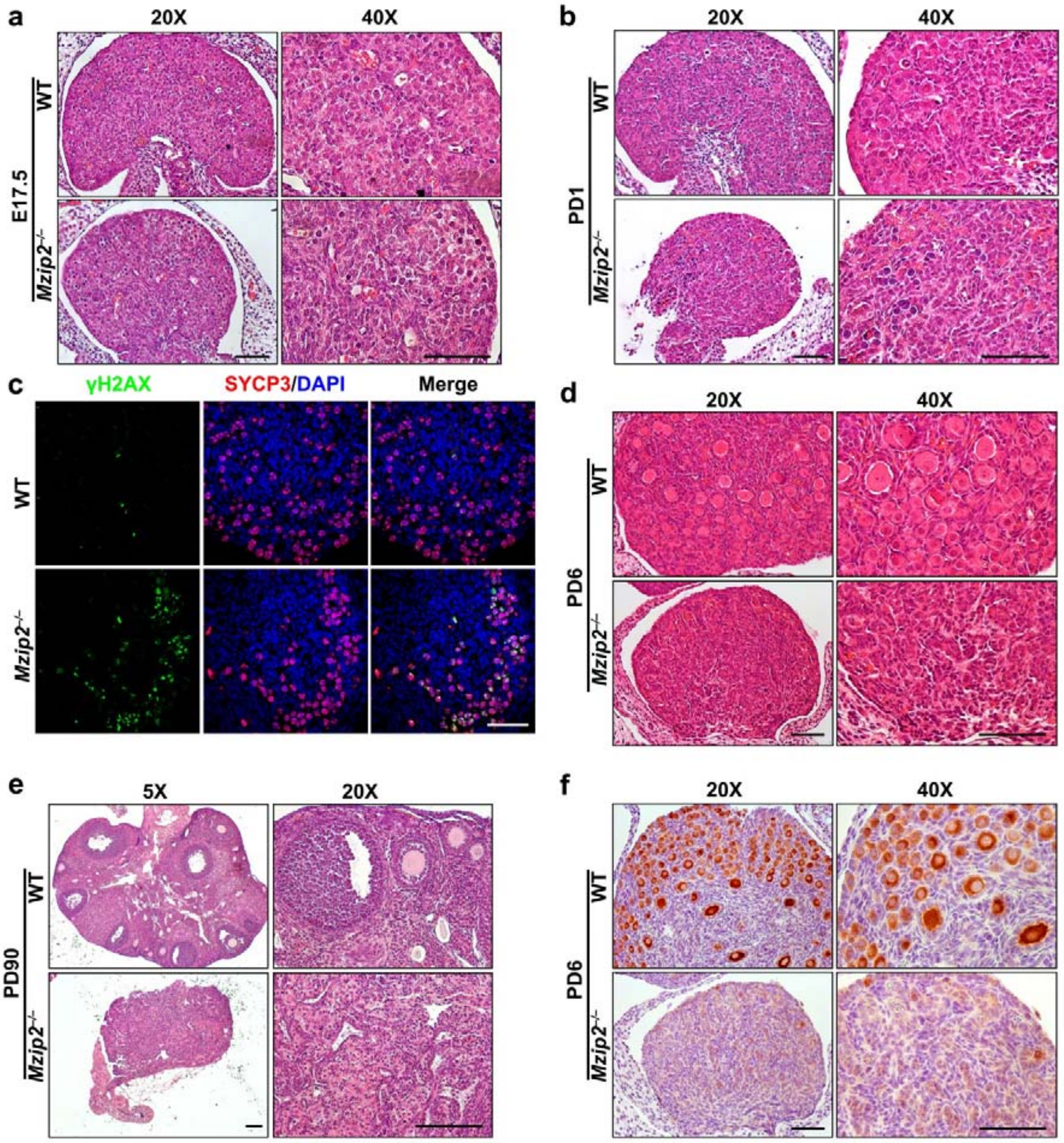

Supplementary Figure 5. Massive oocyte loss in *Mzip2*$^{-/-}$ ovaries. a–b, Histology of WT and *Mzip2*$^{-/-}$ ovaries at E17.5 (**a**) and PD1 (**b**) was shown. Scale bars, 50 μm. **c,** γH2AX (green) staining on sections of WT and *Mzip2*$^{-/-}$ ovaries at E17.5. Scale bar, 50 μm. **d–e**, Histology of WT and *Mzip2*$^{-/-}$ ovaries at PD6 (**d**) and PD90 (**e**) were shown. Scale bars, 50 μm for panel (**d**) and 100 μm for panel (**e**). **f**, Immunohistochemistry (IHC) staining of MVH (mammalian homolog of vasa) showing the oocytes in WT and *Mzip2*$^{-/-}$ ovaries at PD6. Scale bars, 50 μm.

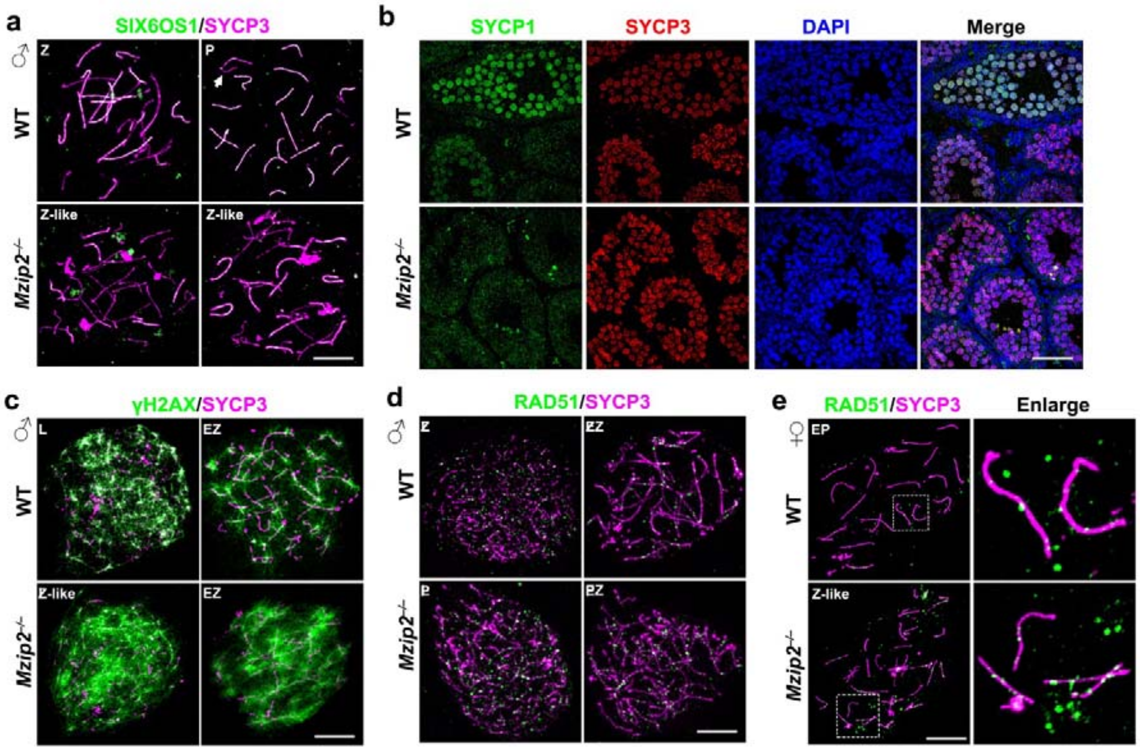

Supplementary Figure 6. Meiotic prophase I progression in meiocytes null for MZIP2. a, Staining of SIX6OS1 on WT and *Mzip2*$^{-/-}$ spermatocytes showing the synapsis progression. Scale bar, 10 µm. **b**, Staining of SYCP1 on slides prepared with WT and *Mzip2*$^{-/-}$ testes. Scale bar, 50 µm. **c**, γH2AX (green) staining on nuclear surface spreads of WT and *Mzip2*$^{-/-}$ spermatocytes at indicated stages. L, leptonema; EZ, early-zygonema. Scale bar, 10 µm. **d,** Staining of RAD51 on WT and *Mzip2*$^{-/-}$ spermatocytes. Scale bars, 10 µm. **e,** RAD51 was stained on the nuclear surface spreads of WT and *Mzip2*$^{-/-}$ oocytes at E17.5. Scale bars, 10 µm.

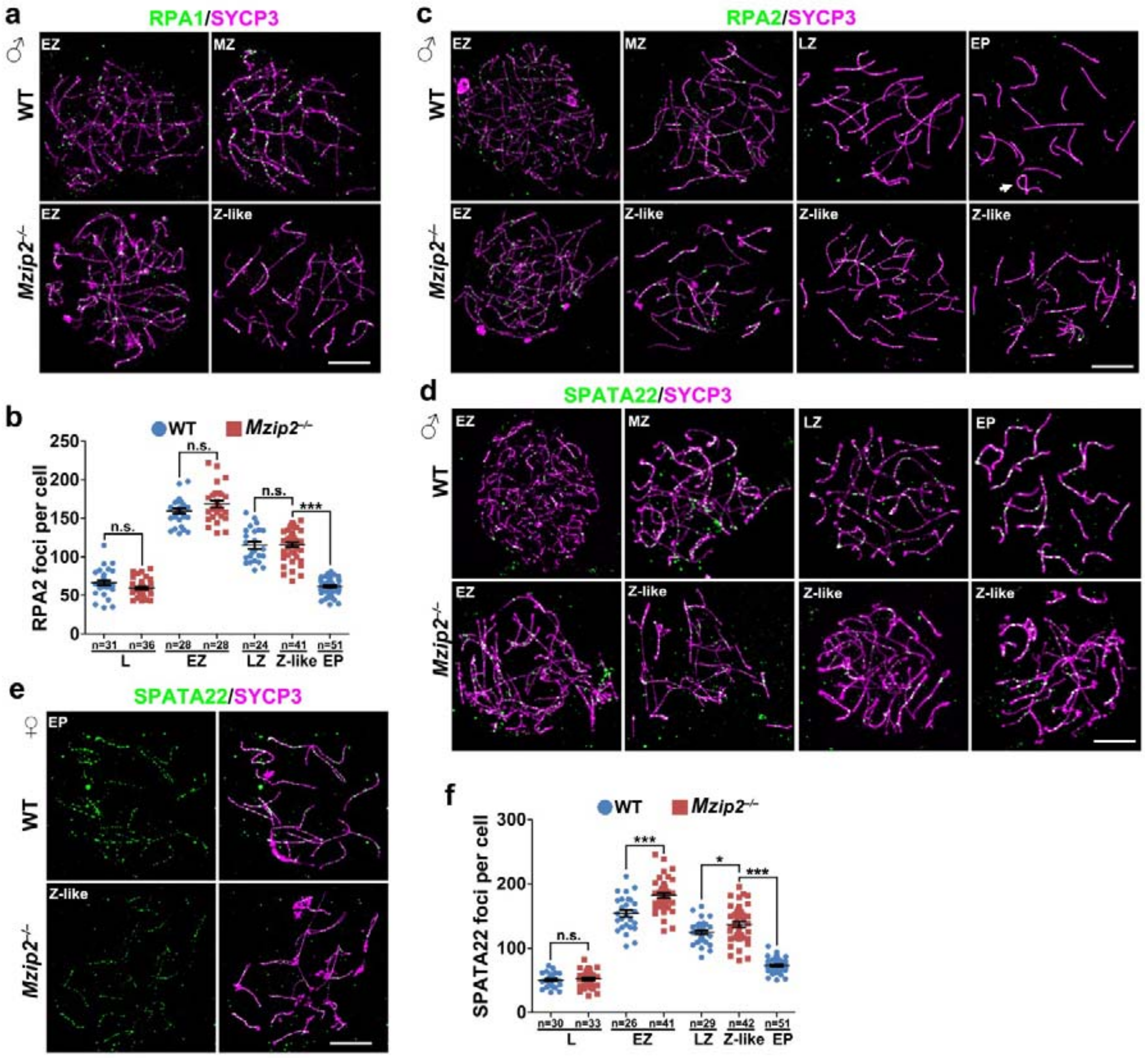

Supplementary Figure 7. Foci of RPA complex in meiocytes null for MZIP2. a, Staining of RPA1 on WT and *Mzip2*$^{-/-}$ spermatocytes. Scale bars, 10 μm. (**b–c**) Staining of RPA2 on nuclear surface spreads prepared from WT and *Mzip2*$^{-/-}$ PD42 testes (**c**), and the quantification of RPA2 foci (**b**). Scale bars, 10 μm. **d–f**, Staining of SPATA22 on nuclear surface spreads prepared from WT and *Mzip2*$^{-/-}$ PD42 testes (**d**) and E17.5 ovaries (**e**), and the quantification of SPATA22 foci (**f**). Scale bars, 10 μm.

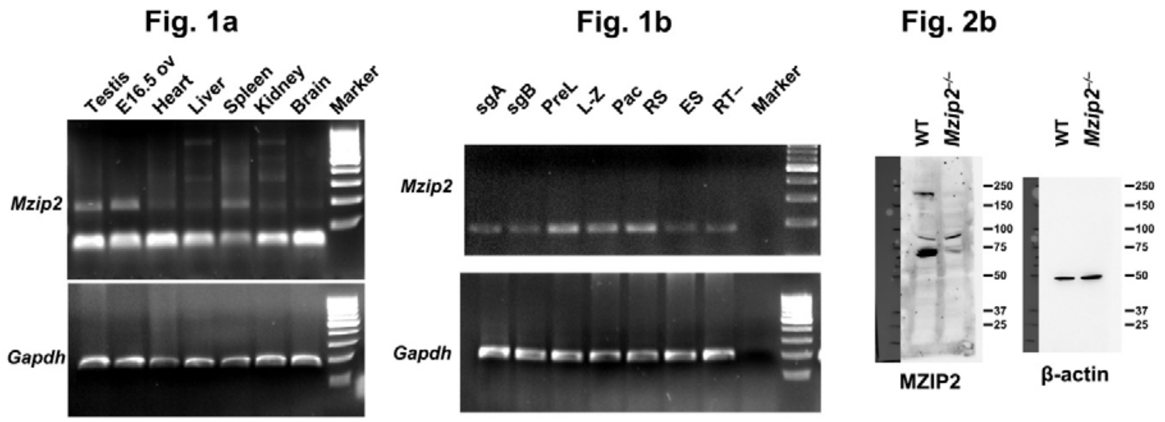

Supplementary Figure 8. Uncropped images of DNA gels and Western blots presented in Fig. 1 and Fig. 2.

Supplementary Table 1. The distribution of *Mzip2*$^{+/+}$, *Mzip2*$^{+/–}$ and *Mzip2*$^{–/–}$ pups from *Mzip2*$^{+/–}$ and *Mzip2*$^{+/–}$ breeding.

Genotype	+/+ (%)	+/– (%)	–/– (%)	Total
♂+/– X ♀+/–	**55** (24.4%)	**117** (52%)	**53** (23.6%)	**225**

Supplementary Table 2. Fertility test of indicated breeding. * no pups observed through a 3-month fertility test. n > 6 for each breeding set.

Genotype	Pups/litter	Litters
♂ +/– X ♀ +/–	7.74 (±0.31)	42
♂ +/– X ♀ –/–	0*	0
♂ –/– X ♀ +/–	0*	0

Supplementary Table 3. Comparisons of the phenotypes in the complete loss mouse model (this study) and the hypomorphic mouse model.

	**Complete deletion in this study**	**Hypomorph model (Guiraldelli et al, 2018)**
**Deletion of protein**	Complete deletion	Remaining functional protein
**Testes weight**	26 mg	50 mg
**Female fertility**	Infertile	Normal fertility
**Arrest stage (spermatogenesis)**	Zygotene-like	Metaphase
**DSB repair (spermatocytes)**	Not repaired	Major repair
**Synaptic defects (spermatocytes)**	Severe	Mild
**TEX11 foci (spermatogenesis)**	Totally abolished	Not determined
**MSH4 foci (spermatogenesis)**	Totally abolished	Not determined
**MLH1 foci (spermatogenesis)**	Totally abolished	Decreased

Supplementary table 4. Primer sequences.

**Primer name**	**Genes targeted**	**Application**	**Sequences (5'-3')**
P1	*Mzip2*	Genotyping (435bp for WT)	5'-TTGCATTACTTTCAAGTGGTGG-3'
P2			5'-GCTCTGGACTTTCAATGACATTAC-3'
P3	*Mzip2*	Genotyping (with P1; 240bp for WT allele)	5'-CGTTCTCTATTCCAGGTGGAGA-3'
P4	*Mzip2*	Genotyping (with P1; 240bp for null allele)	5'-TTATCGTTCTCTATTCCAGGTTTG-3'
S519	*Spo11*	Genotyping (165bp/200bp for WT/null allele)	5'-CTGCTCAGGGAGGAGAACAC-3'
S520			5'-TCAGGACAGGGCATAGCAGT-3'
S521			5'-GCCAGAGGCCACTTGTGTAG-3'
S235	*Mzip2*	RT-PCR (184bp)	5'- ATTCACTTGCAGATATGAGGAACA-3'
S236			5'- AAGATCTCCAGCTTAGTCTCCTGA-3'
Z531	*Gapdh*	RT-PCR (181bp)	5'-ACACTGAGGACCAGGTTGTCTC-3'
Z532			5'-TACTCCTTGGAGGCCATGTAG-3'

Supplementary Table 5. Antibody information.

**Protein name**	**Manufacture (catalogue number)**	**Origin**	**Applications (working dilution)**	**Website Link***
MZIP2	homemade	Rabbit	WB (1:500); IF (1:100)	Immunogen: aa474-635 of mouse MZIP2; Immunized by Core facilities, Zhejiang University School of Medicine.
SYCP1	Abcam (ab15087)	Rabbit	IF (1:200)	https://www.citeab.com/antibodies/771942-ab15087-anti-scp1-antibody
SYCP3	homemade	Rat	IF (1:500)	Immunogen: Full length of mouse SYCP3
SIX6OS1	homemade	Rabbit	IF (1:200)	Immunogen: aa1-200 of mouse SIX6OS1; Immunized by Capra Science Antibodies AB.
RAD51	Abcam (ab176458)	Rabbit	IF (1: 200)	http://www.abcam.com/rad51-antibody-chip-grade-ab176458.html
RPA1	Abcam (ab87272)	Rabbit	IF (1: 50)	http://www.abcam.com/rpa70-antibody-ab87272.html
RPA2	Cell Signaling (2208T)	Rat	IF (1: 100)	https://www.cellsignal.com/products/primary-antibodies/rpa32-rpa2-4e4-rat-mab/2208
SPATA22	Proteintech (16989-1-AP)	Rabbit	IF (1: 100)	http://www.ptglab.com/products/SPATA22-Antibody-16989-1-AP.htm
MLH1	BD (551092)	Mouse	IF (1: 200)	http://www.bdbiosciences.com/us/applications/research/apoptosis/purified-antibodies/purified-mouse-anti-mlh-1-with-control/p/551092
MSH4	Abcam (ab58666)	Rabbit	IF (1: 50)	http://www.abcam.com/msh4-antibody-ab58666.html
MVH	Abcam (ab13840)	Rabbit	IF (1: 200); IHC (1: 400)	http://www.abcam.com/ddx4--mvh-antibody-ab13840.html

Protein name	Manufacture (catalogue number)	Origin	Applications (working dilution)	Website Link*
γH2AX	Cell Signaling (9718S)	Rabbit	IF (1: 400)	https://www.cellsignal.com/products/primary-antibodies/phospho-histone-h2a-x-ser139-20e3-rabbit-mab/9718?N=4294956287&Ntt=h2a.x&fromPage=plp
Cleaved caspase 3	Cell Signaling (9664S)	Rabbit	IF (1: 200)	https://www.cellsignal.com/products/primary-antibodies/cleaved-caspase-3-asp175-5a1e-rabbit-mab/9664
TRF1	homemade	Mouse	IF (1:200)	Immunogen: Full length of mouse TRF1
HORMAD 1	Abcam (ab155176)	Rabbit	IF (1: 200)	http://www.abcam.com/hormad1-antibody-ab155176.html
CREST	Fitzgerald Industries (70R-21494)	Human	IF (1: 200)	https://www.fitzgerald-fii.com/crest-antibody-70r-21494.html
PLZF	Santa Cruz (sc-22839)	Mouse	IF (1:200)	https://www.scbt.com/scbt/sv/product/plzf-antibody-h-300?requestFrom=search
